# COVID-19-Induced Graves’ Disease

**DOI:** 10.7759/cureus.22260

**Published:** 2022-02-15

**Authors:** Hagop Ghareebian, Cary Mariash

**Affiliations:** 1 Internal Medicine/Endocrinology Division, Indiana University, Indianapolis, USA

**Keywords:** thyrotoxicosis, autoimmunity, thyroid, sars covid2, covid-19, graves’ disease

## Abstract

COVID-19, a multi-system disease, could potentially play a role in thyroid dysfunction. New reports show a prevalence of COVID-related thyroiditis. Recent studies suggest that there may be a higher risk of thyroiditis in the setting of SARS-CoV-2, and several cases of Graves’ disease have been reported in individuals with SARS-CoV-2, although the incidence of such findings and their relationship to COVID-19 is unknown. In this report, we present Graves’ hyperthyroidism in a 48-year-old African American male who was admitted to the hospital for complaints of cough, fatigue, and palpitations. He tested positive for SARS-CoV-2 and was found to have suppressed thyroid-stimulating hormone (TSH) and an elevated free T4. The patient had no prior history of thyroid disease. Initially, it was thought to be a case of viral thyroiditis, and he was discharged on prednisone. However, he was found to have positive thyroid-stimulating immunoglobulin (TSI) and a diffuse increase in flow on doppler ultrasound of the thyroid. Subsequently, he was started on anti-thyroid medications with significant improvement. What is unique about this case is that, unlike other described cases in the literature where there was a relapse of a known Graves' disease after COVID-19 disease, our patient did not have a history or symptoms of thyroid disease prior to this event, which should raise the concern about possible activation of Graves' disease after SARS-CoV-2 infection through an autoimmune pathway. In our opinion, physicians, particularly endocrinologists, must be aware of this condition and keep it in mind as a potential differential diagnosis when encountering a similar clinical scenario.

## Introduction

COVID-19, a multi-system disease, could potentially play a role in thyroid disease. New reports show a prevalence of COVID-related thyroiditis. Recent retrospective analysis suggests that there may be a higher risk of thyroiditis in the setting of systemic immune activation due to COVID-19. Additionally, several cases of Graves’ disease have been reported in individuals with the SARS-CoV-2 [1,2], although the incidence of such findings and the relationship to COVID-19 is unknown. In this case, we report Graves’ hyperthyroidism in a COVID-19 patient with no prior history of thyroid disease.

## Case presentation

The patient is a 48-year-old African American male who presented to the emergency department because of a syncopal episode. He reported feeling unwell for the week prior to admission, with symptoms of fatigue, mild shortness of breath, generalized muscle aches, and an occasional cough. He denied fever, chills, abdominal pain, nausea, vomiting, diarrhea, or edema.

An EKG showed a rapid atrial flutter with 4:1 conduction. He reported no prior history of arrhythmias. The patient was converted back to normal sinus rhythm with an amiodarone infusion, and he was subsequently started on flecainide, metoprolol, and apixaban. At the time of initial evaluation, he was found to have positive SARS-CoV-2 testing from a nasal swab. Treatment for COVID-19 with 6 mg of oral dexamethasone daily was started.

On physical examination, initial vital signs showed a heart rate of 120-140, blood pressure of 90/60, a non-tender symmetrical enlargement of the thyroid gland, and no exophthalmos, but there were tremors in both hands and brisk deep tendon reflexes.

**Figure 1:**
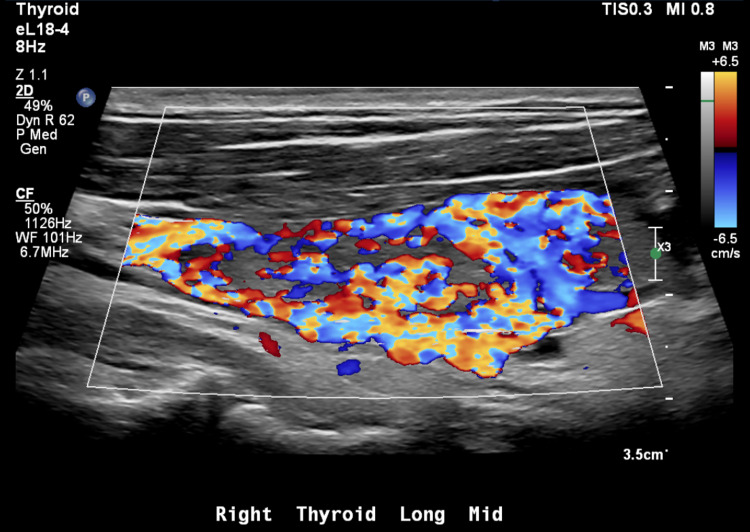
Increased flow of the right thyroid lobe on color doppler ultrasound

**Figure 2:**
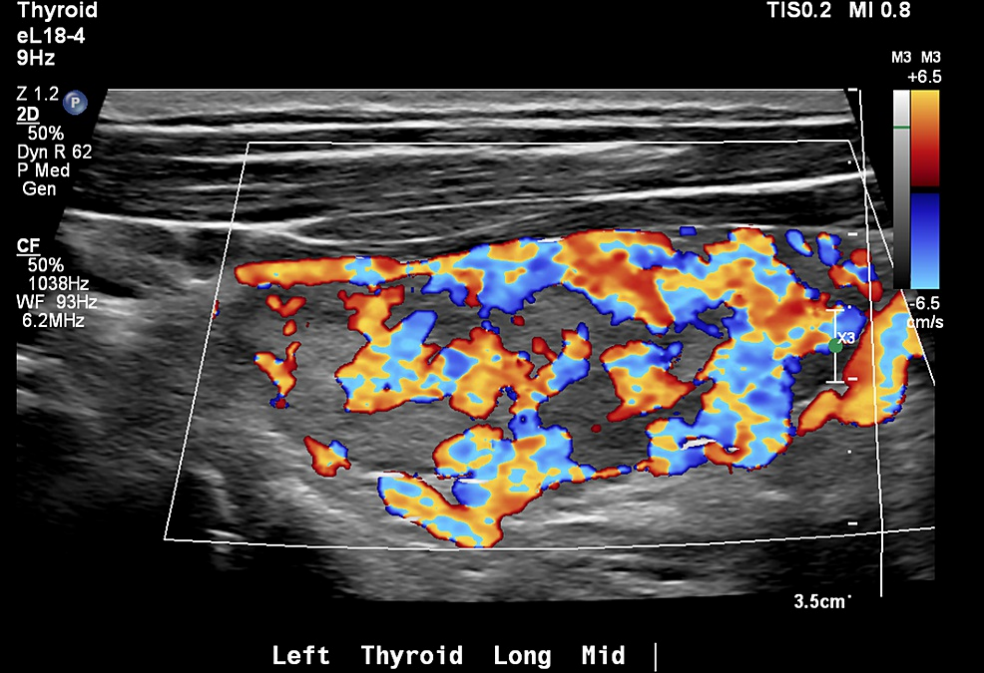
Increased flow of the left thyroid lobe on color doppler ultrasound

The thyroid-stimulating hormone (TSH) was <0.010 mcU/mL (0.400-4.200 mcU/mL) and free T4 was 2.8 (0.6-1.5 ng/dl). Thyroid ultrasound showed a diffusely heterogeneous and irregular thyroid with widely increased flow on color doppler imaging. No nodules were identified (Figures 1-2).

Dexamethasone for COVID-19 is given for a duration of six days, and prednisone 20 mg daily for five days was prescribed upon discharge due to suspected COVID-19 associated thyroiditis. The patient had no prior history of hyperthyroidism or Graves’ disease. Thyroid-stimulating immunoglobulin (TSI) was drawn before hospital discharge. At a follow-up outpatient clinic visit shortly after hospital discharge, it was noted that the TSI was positive at 6.31 (upper normal <0.51). Therefore, a diagnosis of Graves’ disease was made, and prednisone was discontinued. The patient was started on methimazole 10 mg tablets twice a day and metoprolol succinate 25 mg tablets daily. The patient has been following up regularly with the endocrinology clinic.

## Discussion

Genetic, environmental, and immunological factors contribute to the development of autoimmune diseases, including Graves' disease. Viral infections, known triggering environmental factors, are considered to play a fundamental role in the pathogenesis of autoimmune thyroid disease [1]. As opposed to the other reported cases [2,3], our patient had no previous history of either Graves’ disease, thyroid autoimmunity, or other autoimmune phenomena. Our case describes the new onset of Graves’ disease after SARS COV-2 infection. The SARS-CoV-2 utilizes the angiotensin-converting enzyme 2 (ACE2) receptor for its initial entry port into the cells [4]. ACE2 expression was found to be higher in the epithelial cells of the lungs, kidneys, heart, small intestine, blood vessels, adipose tissue, and thyroid gland [5]. SARS-CoV-2 infection may induce new-onset or latent autoimmunity, including autoimmune thyroid disease, but the exact mechanism is still not very clear.

One proposed explanation is that the exaggerated inflammatory response associated with severe COVID-19 disease could have triggered an immunological cascade that activates or reactivates Graves’ disease. The inflammatory response induced by SARS-CoV-2 is mostly led by Th1 cytokines as well as interleukin-6 (IL-6). On the other hand, increased levels of IL-6 in patients with Graves’ disease have been reported in the pathogenesis of the disease [6].

Additionally, new data indicate the important role of novel Th lymphocyte subsets (e.g., Th17, Th22) and their cytokines in autoimmune thyroid disease pathogenesis [7]. SARS-CoV-2 infection-associated hyper inflammation could trigger Th17 and the autoimmune thyroid disease as evidenced by another study [8]. Finally, various vaccines' adjuvants were reported to turn on different unfavorable immune responses that result in a spectrum of autoimmune disorders including thyroid diseases [9].

## Conclusions

As evidenced by recent studies, the SARS-CoV-2 infection could cause Graves’ disease. Different autoimmune factors may mediate intricate pathogenic signaling in autoimmune hyperthyroidism. Future studies are needed to rule out the autoimmune factors and molecular pathways involved in SARS-CoV-2-induced autoimmune hyperthyroidism. The direct relationship between COVID-19 and the thyroid gland is rapidly evolving. This case may alert clinicians to the careful observation of this association and is an important differential diagnosis to keep in mind when patients present with hyperthyroidism post-COVID-19 disease.
